# Evaluation of the Effect of Topically Applied Melatonin and Vitamin C in the Non-surgical Treatment of Chronic Periodontitis: A Triple-Blind Randomized Clinical Trial

**DOI:** 10.7759/cureus.76676

**Published:** 2024-12-31

**Authors:** Kani M Mohamad Rauf, Ali F Alzubaidee, Nasreen H Hamonari

**Affiliations:** 1 Dental Public Health, Kurdistan Higher Council of Medical Specialties, Erbil, IRQ; 2 Oral Medicine, Kurdistan Higher Council of Medical Specialties, Erbil, IRQ; 3 Community Medicine/Preventive Dentistry, Dental Public Health Center, Kurdistan Higher Council of Medical Specialties, Erbil, IRQ

**Keywords:** chronic periodontitis, melatonin, non-surgical periodontal therapy, periodontal pocket, topically applied drug, vitamin c

## Abstract

Aim: This study aimed to evaluate the impact of using melatonin and vitamin C as adjuncts to the non-surgical treatment of chronic periodontitis.

Materials and methods: This triple-blind randomized clinical trial involved 100 participants with chronic periodontitis. Subjects were randomly assigned to three groups: (1) non-surgical periodontal therapy (NSPT) alone (n = 33); (2) NSPT with melatonin (n = 33); and (3) NSPT with melatonin and vitamin C (n = 34). Clinical parameters, including gingival index (GI), probing depth (PD), and clinical attachment loss (CAL), were assessed at baseline and at one week, one month, and three months post treatment. Statistical significance was set at p ≤ 0.05.

Results: Compared to baseline, all groups showed significant improvements in periodontal parameters (p ≤ 0.001). At three months, the group receiving both melatonin and vitamin C demonstrated the greatest reduction in PD (mean reduction: 1.96 mm, p ≤ 0.001) and CAL (mean reduction: 1.87 mm, p ≤ 0.001). This group also achieved the most significant improvement in GI (mean reduction: 1.74, p ≤ 0.001).

Conclusions: The combined adjunctive therapy of melatonin and vitamin C demonstrated superior improvements in periodontal indices compared to NSPT alone, supporting its potential as an effective adjunctive treatment for chronic periodontitis.

## Introduction

Periodontitis is a chronic inflammatory condition characterized by progressive destruction of the supporting structures of teeth due to an imbalance between oxidant and antioxidant systems [1]. Melatonin, recognized as an antioxidant biomarker, plays a crucial role in combating oxidative stress. This oxidant-antioxidant imbalance is closely linked to the development and progression of periodontal disease [2]. Animal studies have demonstrated that melatonin, beyond its antioxidant properties, also exhibits anti-inflammatory and anti-cancer effects [3].

Periodontal diseases affect a significant portion of the global population, with prevalence rates reaching 10-15%. Chronic periodontitis, a specific form of periodontal disease, targets the tissues surrounding and supporting teeth, leading to the progressive destruction of connective tissue and bone [1]. This condition is characterized by the formation of periodontal pockets, gingival recession, and, if left untreated, eventual tooth loss [4]. Due to its high worldwide prevalence and substantial impact on oral health and quality of life, chronic periodontitis has been recognized as a major public health concern [5].

Non-surgical periodontal therapy (NSPT) is the gold standard for managing periodontitis by mechanically removing plaque and calculus on the root surface [6]. However, restrictions imposed by the mobility of the tooth and immune suppression may impede tooth regeneration [7]. While NSPT has shown efficacy in reducing inflammation and managing progressive bone loss in some cases, the periodontium's capacity to entirely restore its previous structure and functionality remains limited [8]. Regarding these issues, periodontists have contemplated utilizing host modulatory drugs as supplementary therapies to regulate the host immune response and enhance the regeneration of periodontal tissues [9]. Host modulatory agents can influence the host's immune reply through pharmacological mechanisms [10,11]. Treatment of periodontal disease aims to prevent the disease's progression and regenerate the lost supporting structures [12].

Melatonin (N-acetyl-5-methoxytryptamine) is an indole amine synthesized in the pineal gland, which exhibits a variety of biological activities, including antioxidant, anti-inflammatory, and immunomodulatory effects [13,14]. Furthermore, melatonin has been reported to enhance bone differentiation and promote osteoblast formation [15]. Melatonin is a free radical scavenger in both pharmacological and physiological blood concentrations [16]. The findings showed that melatonin levels in blood and saliva are significantly reduced in those diagnosed with periodontitis compared to those considered normal [17].

Vitamin C or L-ascorbic acid is a hydrophilic vitamin with antioxidative and anti-inflammatory characteristics. Collagen formation and wound healing are of significant importance [18,19]. Several studies in periodontitis have shown that ascorbic acid application, either locally or as a supplement, has a significant preventive and therapeutic effect on gingival health [20]. The study aims to investigate the effect of the topical application of melatonin and vitamin C as adjunctive use in the non-surgical treatment of chronic periodontitis.

## Materials and methods

Study design and setting

This triple-blind randomized clinical trial was conducted at Azadi Dental Center, Erbil, Kurdistan Region, Iraq, from August 1, 2023, to August 1, 2024.

Participants

Sample Size Calculation

The sample size was calculated using Kelsey's formula for clinical trials, assuming a medium effect size, a power of 80%, and a significance level of 5% [21].

$n=(Z^2*p*(1-p))/e^2$

The calculation determined a minimum sample size of 60 participants, which was increased to 100 to account for potential losses during follow-up.

Patient Enrollment and Randomization

Eligible participants were screened and enrolled by trained research assistants at Azadi Dental Center, Erbil, based on predefined inclusion and exclusion criteria. Informed consent was obtained from all participants before inclusion.

Randomization was performed using a computer-generated randomization sequence, and block randomization ensured balanced allocation across the three groups. The random allocation sequence was concealed using opaque, sealed envelopes prepared by an independent assistant. These envelopes were opened only after participant enrollment.

Blinding

This study employed a triple-blind design to prevent bias. The participants, examiner, and statistician were blinded to group assignments. Identical placebo and active treatments ensured effective blinding during the intervention phase.

Data collection

**Figure 1:**
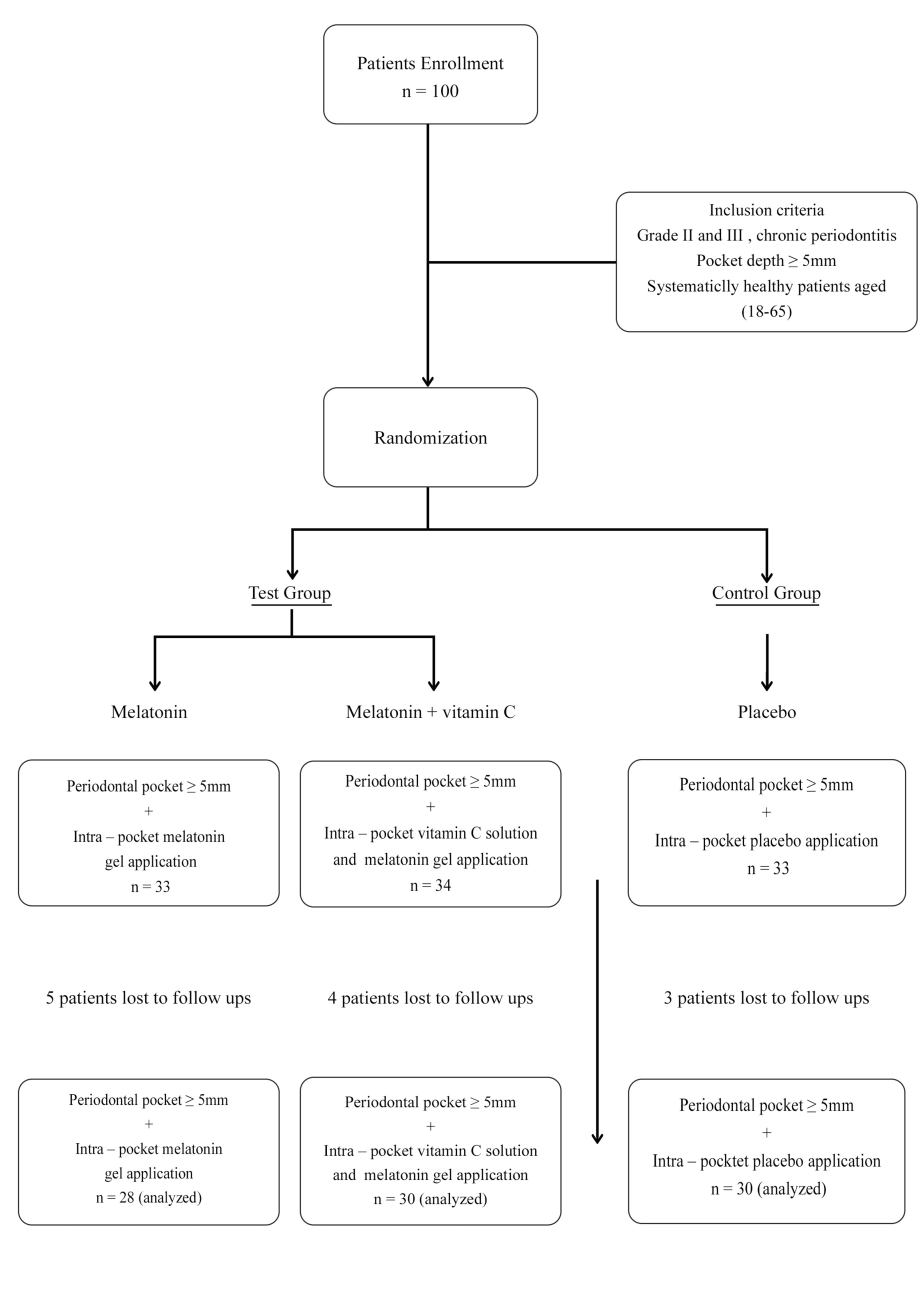
Flowchart of the study.

The study focused on patients with localized chronic periodontitis, stages II and III (as defined by the 2017 World Workshop of Periodontology) [22], based on clinical criteria [23]. Participants were aged 18-65 years with pocket depths ≥5 mm [21]. The initial sample size of 100 was determined using a questionnaire that collected sociodemographic data [21]. Due to attrition, the final sample comprised 88 patients with 49 females and 39 males (Figure 1). Only healthy individuals were included [21]. Exclusion criteria encompassed pregnant and lactating women, smokers, and those who had used non-steroidal anti-inflammatory drugs, mouthwash, or vitamin supplements within three months prior to the study [21]. Participants provided informed consent and received instructions on proper oral hygiene techniques, including modified Bass technique for brushing and flossing twice daily. They were also advised to avoid medications that could interfere with the trial.

Pilot study

A pilot study was conducted in August 2023 to ensure data consistency through intra- and inter-examiner calibration, involving 10 patients. The examination protocol included three key measurements: the gingival index (GI) of Loe and Silness (1963) [21] and the periodontal disease indices (PDI) of Ramfjord (1959), which comprised probing depth (PD) [21] and clinical attachment loss (CAL) [21].

Inter-examiner calibration was performed by having both the researcher and a specialist examine the patients independently. For intra-examiner calibration, the researcher examined the patients twice, with a two-hour interval between examinations.

Shapiro-Wilk test showed that the GI and CAL distributions were normal, while PD was not normal. No significant differences were detected between the researcher and the specialist readings regarding GI (p = 0.115), PD (p = 0.157), and CAL (p = 0.112).

Clinical examinations

The intra-oral examination assessed gingival inflammation using the GI, as well as measuring PD and CAL. The PD was measured from the pocket base to the gingival crest, while CAL was measured from the pocket base to the cemento-enamel junction [21]. These measurements were taken using William's periodontal probe (MEDESY, Maniago, Italy) [23]. For each index tooth, measurements were recorded at four surfaces: mid-buccal/facial, mid-lingual/palatal, mesiobuccal, and distobuccal [21].

Drug composition and preparation

Placebo Oral Gel Preparation

One percent of the placebo gel consisted of 1% carboxymethyl cellulose in distilled water alone (1 gm carboxymethyl cellulose mixed in 100 ml of distilled water by continuous manual mixing) [11]. Carboxymethyl cellulose = carboxymethyl cellulose sodium salt, low viscosity 9004-32-4.

Melatonin Oral Gel Preparation

Five percent of melatonin gel was prepared at a neutral pH (7.5) from 5 gm of pure melatonin powder added to the base (1% carboxymethyl cellulose in distilled water) with the aid of a mixer machine [11]. Melatonin gel was stored at 18-25°C temperature to give high viscosity and better release of active agent [24]. Melatonin powder = 73-31-4 (Mediver, London, UK).

Vitamin C Solution Preparation

Vitamin C was prepared from vitamin C powder. Ten grams of this powder was dissolved in 50 ml of distilled water by continuous manual mixing to prepare a 50 ml solution [25]. Vitamin C solution was stored at a temperature between 2°C and 8°C and protected from light until the time of use [26]. Vitamin C powder = L-ascorbic acid 99.5%, BP, Eur., USP grade (Mediver).

The preparation procedure was conducted at the College of Pharmacy, Department of Pharmaceutical Chemistry.

Periodontal therapy

Participants were randomly divided into three groups using computer software: placebo, melatonin, and melatonin with vitamin C [21]. Initial clinical parameters were recorded pre-treatment. All participants received full mouth scaling and root planing (SRP) using an ultrasonic device (DTE, D2 LED, Guilin Woodpecker Medical Instrument Co. Ltd., Guilin, China) [23] and Gracey periodontal curettes (Hu-Friedy Instruments, Chicago, IL, USA) [11,27], followed by coronal polishing [23].

Patients returned after seven days for local drug application, which continued weekly for four weeks [11,23]. The placebo group (33 patients) received 1 ml of 1% placebo gel. The melatonin group (33 patients) received 1 ml of 5% melatonin gel. The melatonin and vitamin C group (34 patients) received 1 ml of 250 mg vitamin C liquid for five minutes, followed by 1 ml of melatonin gel [25]. The application was done using disposable syringes with blunt needles [23].

Patients were instructed not to rinse or eat for 30 minutes after application [25]. A dental assistant managed daily arrangements to maintain researcher blindness.

Clinical measurements were taken four times: pre-treatment (baseline) [11,21], one week post therapy [26], one month post intervention [25], and three months post treatment [27]. Measurements included GI, CAL, and PD. A single examiner (the researcher) conducted all clinical examinations. The researcher, participants, and statistician were blinded to treatment types.

Ethical considerations

This study was submitted to the Ethics and Scientific Committee at Kurdistan Higher Council of Medical Specialties and obtained ethical approval (number: 560) on 2023/2/14. The study protocol was explained carefully and consent was obtained from each patient through a consent form. Confidentiality and anonymity of data were ensured. This study was registered at the ISRCTN registry (ISRCTN68604984; 08.10.2024; https://www.isrctn.com/ISRCTN68604984).

Data management and statistical analysis

Data were recorded on a custom questionnaire, entered into Microsoft Excel 2016 (Microsoft Corporation, Redmond, WA), and analyzed using SPSS version 26 (IBM Corp., Armonk, NY). Statistical methods included a chi-square test to compare proportions across the three study groups, with Fisher's exact test used when expected frequencies were low. Shapiro-Wilk test was used to check data normality, determining the use of non-parametric tests when appropriate. Kruskal-Wallis test was used to compare mean ranks across the three groups, followed by Bonferroni post-hoc tests for pairwise comparisons. Related samples Friedman's two-way ANOVA by ranks was used to compare readings at different time periods within each group, with post-hoc tests for pairwise time period comparisons. A p-value of ≤ 0.05 was considered statistically significant.

## Results

Table 1 shows that 28.4% (25) of the patients were aged less than 30 years, 39.8% (35) were aged 30-49 years, and 31.8% (28) were aged ≥ 50 years, but there was no significant difference between the groups (p = 0.373). More than half (60.2%, 53) of the patients were females, but the difference was not significant (p = 0.607) between the groups.

**Table 1:** Age and sex distribution of patients. * By chi-square test.

Age (years)	G1 - Placebo	G2 - Melatonin Gel	G3 - Vitamin C and melatonin gel	Total	P-value*
<30	6 (20.0)	9 (32.1)	10 (33.3)	25 (28.4)	
30-49	11 (36.7)	10 (35.7)	14 (46.7)	35 (39.8)	
≥50	13 (43.3)	9 (32.1)	6 (20.0)	28 (31.8)	0.373
Sex					
Male	13 (43.3)	9 (32.1)	13 (43.3)	35 (39.8)	
Female	17 (56.7)	19 (67.9)	17 (56.7)	53 (60.2)	0.607
Total	30 (100.0)	28 (100.0)	30 (100.0)	88 (100.0)	

Before the intervention, there was a significant difference between the three study groups regarding the GI (p = 0.022), PD (p = 0.004), and CAL (p = 0.024) readings. This difference is mainly attributed to the difference in the pocket depth measurements between the three groups. Regarding GI, at times 2, 3, and 4, there was no significant difference between the three groups.

Regarding pocket depth, there was a significant difference between the groups in time 2 (p = 0.013), time 3 (p < 0.001), and time 4 (p < 0.001). In time 4, the least PD was observed in group 3 (G3). The difference was significant between G3 and group 1 (G1) (p < 0.001) and between G3 and group 2 (G2) (p < 0.001).

Results showed that there were significant differences between the three groups in CAL readings, starting from time 1 (p = 0.024), time 2 (p = 0.024), and time 4 (p < 0.001). It is evident in Table 2 that the highest readings of CAL before the intervention were in G3, and the lowest readings of CAL three months after the intervention were in G3 (Tables 2, 3).

**Table 2:** Comparing the studied variables of the three study groups at different times of the study. * By Kruskal-Wallis test. ** Reading 1: First reading before the intervention. Reading 2: One week after the intervention. Reading 3: One month after the intervention. Reading 4: Three months after the intervention. GI: gingival index; PD: probing depth; CAL: clinical attachment loss.

Variable time**	G1 - Placebo			G2 - Melatonin gel			G3 - Vitamin C and melatonin			P*
	Mean	Median	Mean rank	Mean	Median	Mean rank	Mean	Median	Mean rank	
GI-1	2.08	2.13	34.72	2.44	2.41	52.84	2.33	2.30	46.50	0.022
GI-2	1.89	2.00	34.63	2.18	2.08	50.39	2.11	2.20	48.87	0.032
GI-3	1.29	1.33	45.77	1.26	1.18	45.45	1.28	1.08	42.35	0.850
GI-4	0.79	0.75	50.98	0.69	0.60	46.04	0.59	0.50	36.58	0.085
PD-1	2.89	2.75	34.70	3.37	3.50	56.50	3.06	3.25	43.10	0.004
PD-2	2.88	2.75	36.88	3.29	3.25	55.82	2.94	3.00	41.55	0.013
PD-3	2.20	2.18	49.13	2.41	2.38	57.07	1.72	1.50	28.13	<0.001
PD-4	1.82	1.75	58.75	1.79	1.88	53.20	1.10	1.00	22.13	<0.001
CAL-1	1.98	1.80	37.30	2.08	2.25	41.45	2.66	3.00	54.55	0.024
CAL-2	1.98	1.80	37.30	2.08	2.25	41.45	2.66	3.00	54.55	0.024
CAL-3	1.95	1.80	41.73	1.91	2.00	41.88	2.23	2.25	49.72	0.385
CAL-4	1.79	1.68	61.08	1.27	1.38	45.93	0.79	0.88	26.58	<0.001

**Table 3:** Post-hoc test comparing two groups in each of the time periods. * N/A (not applicable): The p-value was not calculated by the post-hoc test because the p-value (of the Kruskal-Wallis test) was not significant. ** G1: Placebo. G2: Melatonin gel. G3: Vitamin C and melatonin gel.

Variables	Time 1, p-values	Time 2, p-values	Time 3, p-values	Time 4, p-values
Gingival index (GI)				
**G1 X G2	0.007	0.019	N/A*	N/A*
G1 X G3	0.073	0.031	N/A*	N/A*
G2 X G3	0.344	0.820	N/A*	N/A*
Probing depth (PD)				
G1 X G2	0.001	0.004	0.234	0.399
G1 X G3	0.199	0.476	0.001	<0.001
G2 X G3	0.044	0.032	<0.001	<0.001
Clinical attachment loss (CAL)				
G1 X G2	0.535	0.535	N/A*	0.023
G1 X G3	0.009	0.009	N/A*	<0.001
G2 X G3	0.050	0.050	N/A*	0.004

**Figure 2:**
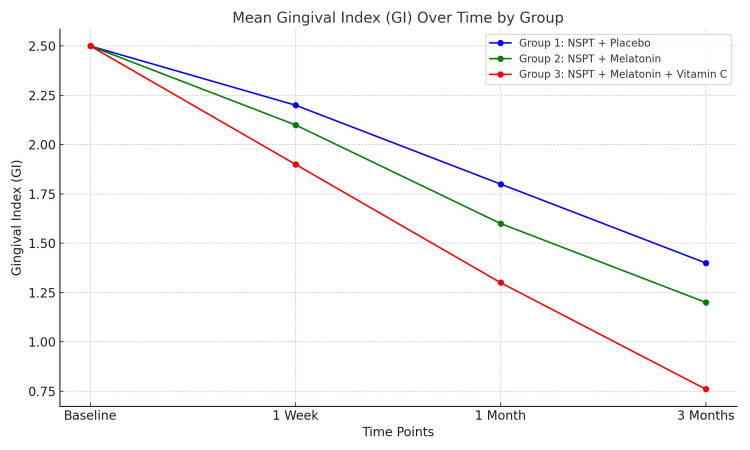
Mean gingival index (GI) over time by groups. This shows the decreasing trend in GI across all groups, with group 3 (melatonin + vitamin C) showing the most significant improvement. NSPT: non-surgical periodontal therapy.

Data points represent mean GI values at baseline, one week, one month, and three months. Error bars indicate standard deviation. Group 3 shows the most substantial reduction over time (p ≤ 0.001) (Figure 2).

The highest means of improvement in GI were in G2 and G3 compared with the placebo. The differences between G2 and G3 with G1 (placebo) were significant (p < 0.001), but there was no significant difference between G2 and G3 (p = 0.836).

**Figure 3:**
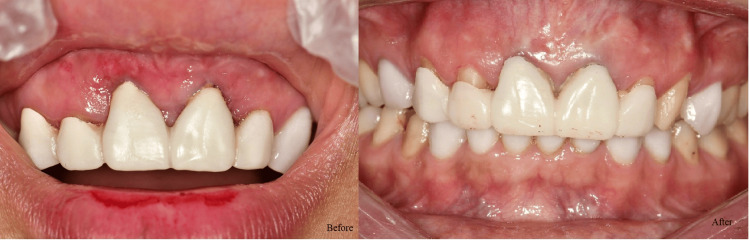
Before: Periodontal disease. After: The effect of local application of vitamin C and melatonin.

**Figure 4:**
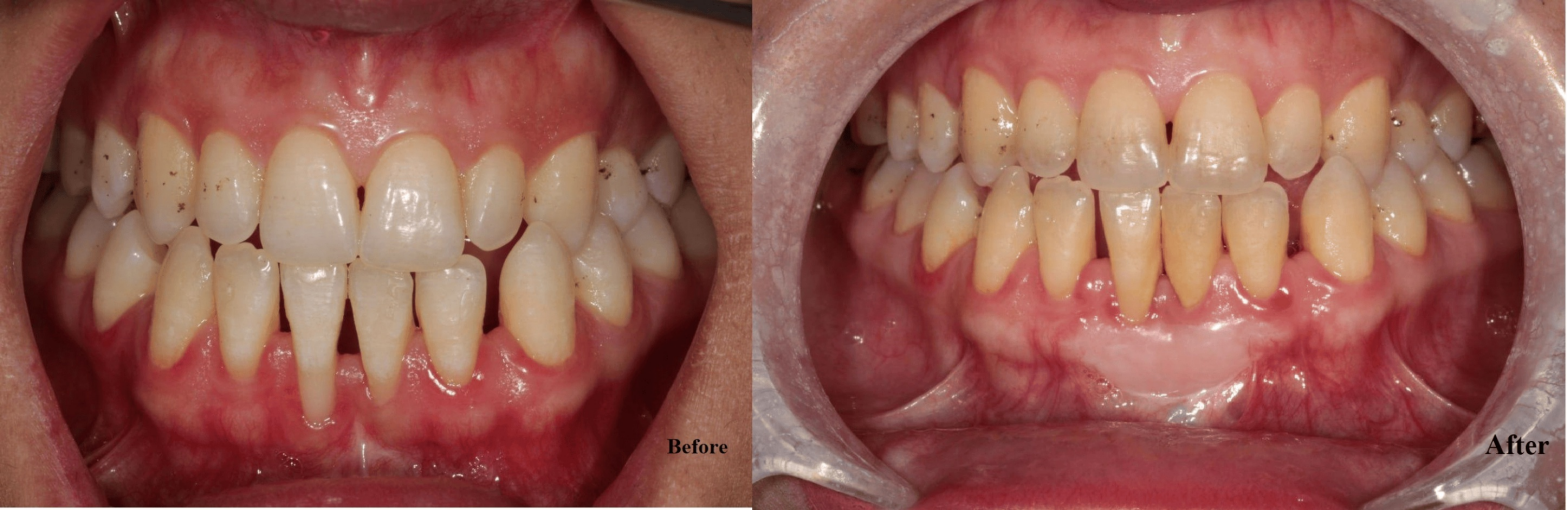
Before: Chronic periodontal case (severe bone loss) in mandibular central incisors. After: The effect of local application of vitamin C and melatonin improved the result of graft in the mandibular anterior region.

**Figure 5:**
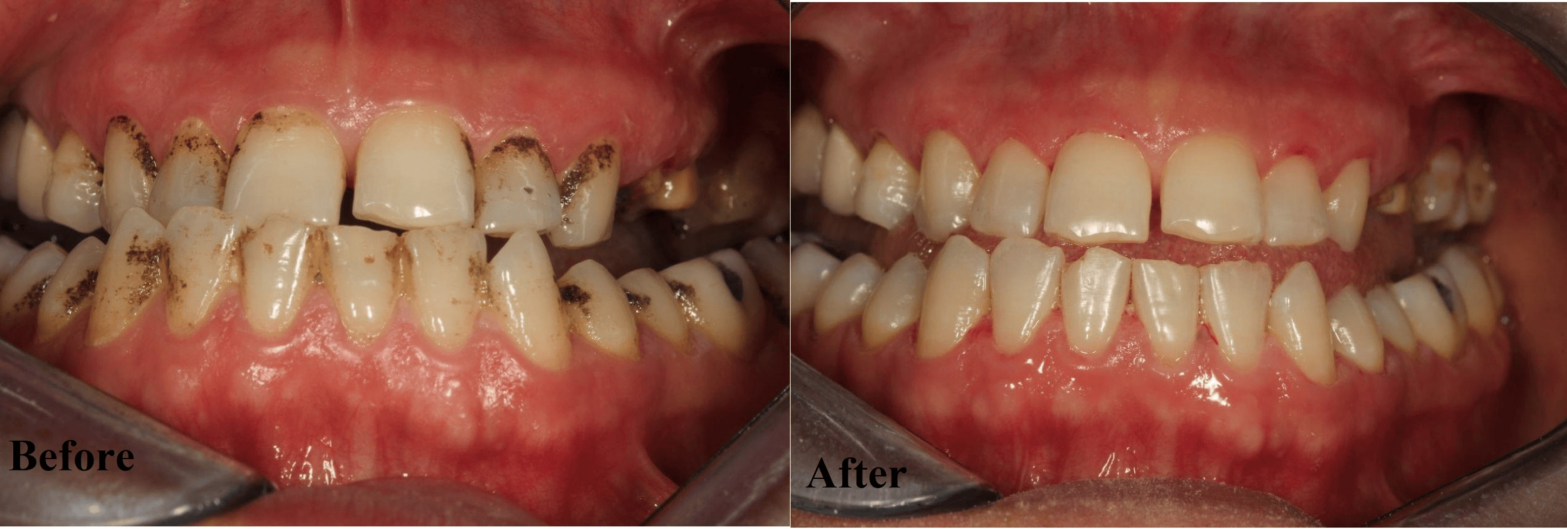
Before: Simple case of periodontitis (periodontal pocket at the maxillary right posterior area). After: The effect of vitamin C and melatonin on the gingiva after full mouth scaling and polishing.

Regarding PD and CAL, the best improvement was noticed in G3, followed by G2 and G1. Across all groups, GI decreased significantly from baseline to three months (p ≤ 0.001). Group 3 (melatonin + vitamin C) achieved the most substantial reduction, with a mean decrease of 1.74 (p ≤ 0.001). Group 2 (melatonin) showed a mean reduction of 1.29, while group 1 (placebo) exhibited the smallest improvement with a mean decrease of 1.08. The intergroup differences were significant at three months (p ≤ 0.001), with group 3 outperforming groups 1 and 2. This indicates a synergistic effect of combining melatonin and vitamin C in improving gingival health. All the differences between the groups were significant (Table 4). Figures 3-5 show the improvement of periodontitis after intervention with vitamin C and melatonin treatment.

**Table 4:** Comparing the mean difference between readings 1 and 4 (mean of improvement) between the three study groups. * Calculated by Kruskal-Wallis test. ** Calculated by a post-hoc test (Bonferroni). GI: gingival index; PD: probing depth; CAL: clinical attachment loss.

Variables	Groups	Mean	SD	Mean rank	P*	Groups	P**
Difference between GI-1 and GI-4 (GI-1 – GI-4)	G1 - Placebo	1.29	0.46	27.32		G1 X G2	<0.001
	G2 - Melatonin gel	1.74	0.40	54.11	<0.001	G1 X G3	<0.001
	G3 - Vitamin C and melatonin gel	1.74	0.43	52.72		G2 X G3	0.836
Difference between PD-1 and PD-4 (PD-1 – PD-4)	G1 - Placebo	1.08	0.49	27.03		G1 X G2	0.005
	G2 - Melatonin gel	1.57	0.72	45.77	<0.001	G1 X G3	<0.001
	G3 - Vitamin C and melatonin gel	1.96	0.62	60.78		G2 X G3	0.024
Difference between CAL-1 and CAL-4 (CAL-1 – CAL-4)	G1 - Placebo	0.19	0.15	17.13		G1 X G2	<0.001
	G2 - Melatonin gel	0.81	0.62	47.54	<0.001	G1 X G3	<0.001
	G3 - Vitamin C and melatonin gel	1.87	0.84	69.03		G2 X G3	0.001

**Figure 6:**
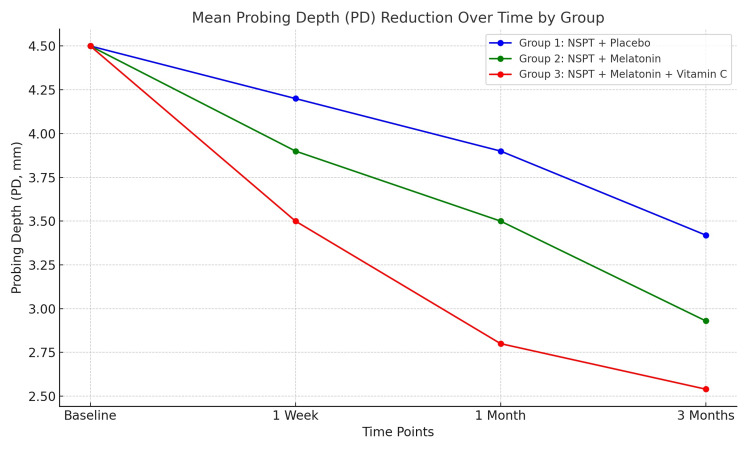
Mean probing depth (PD) reduction over time by group. This demonstrates the progressive reduction in PD, with group 3 showing the greatest reduction compared to groups 1 and 2. NSPT: non-surgical periodontal therapy.

Regarding the comparison of PD changes among groups, group 3 demonstrates the greatest improvement, with significant intergroup differences at three months (p ≤ 0.001) (Figure 6).

**Figure 7:**
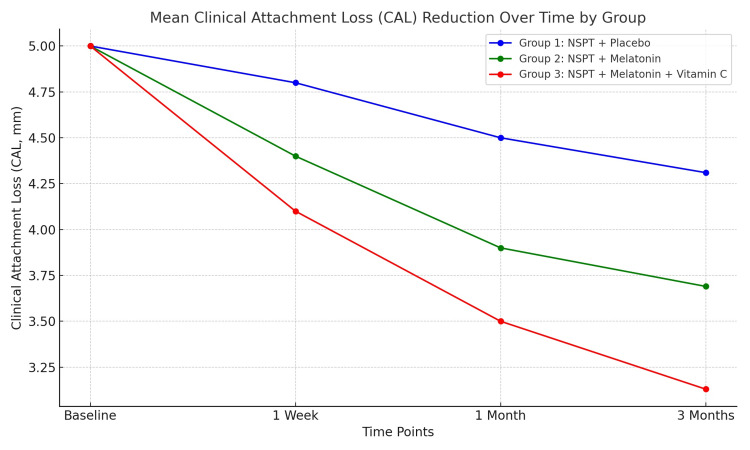
Mean clinical attachment loss (CAL) reduction over time by groups. This reflects the improvement in CAL, again with group 3 achieving the most substantial benefits. NSPT: non-surgical periodontal therapy.

In the control group, the mean, median, and mean rank of GI before the intervention were 2.08, 2.13, and 3.98, respectively (Table 5). There was a consistent and significant decrease in these values, reaching 0.79, 0.75, and 1.03, respectively, three months after the intervention (p < 0.001). All the differences between the different time periods were also significant. The mean, median, and mean rank of PD before the intervention were 2.89, 2.75, and 3.53, respectively, but there was a significant and consistent decrease reaching 1.82, 1.75, and 1.02, respectively, at the end of the study (p < 0.001). Figure 7 highlights CAL improvements across time points. Group 3 significantly outperforms groups 1 and 2 by three months (p ≤ 0.001). The difference between readings 1 and 2 was not significant (p = 0.764), but all the other differences were significant. Regarding CAL, there was also a significant difference between the readings of the four different times (p < 0.001), but the differences between readings 1 and 2 and readings 1 and 3 were not significant (p = 1.000 and p = 0.841, respectively). The difference between readings 1 and 4 was significant (p < 0.001) (Table 5).

**Table 5:** Comparing the studied variables at different time periods in the placebo group. * By related samples Friedman’s two-way ANOVA by ranks. ** By Bonferroni post-hoc test. GI: gingival index; PD: probing depth; CAL: clinical attachment loss.

Variable - Time	Mean	Median	Mean rank	P*	Time	P**	Time	P**
GI-1	2.08	2.13	3.98	<0.001	1 X 2	0.003	2 X 3	0.003
GI-2	1.89	2.00	2.98		1 X 3	<0.001	2 X 4	<0.001
GI-3	1.29	1.33	2.00		1 X 4	<0.001	3 X 4	0.004
GI-4	0.79	0.75	1.03		NA	NA	NA	NA
PD-1	2.89	2.75	3.53	<0.001	1 X 2	0.764	2 X 3	<0.001
PD-2	2.88	2.75	3.43		1 X 3	<0.001	2 X 4	<0.001
PD-3	2.20	2.18	2.02		1 X 4	<0.001	3 X 4	0.003
PD-4	1.82	1.75	1.02		NA	NA	NA	NA
CAL-1	1.98	1.80	2.88	<0.001	1 X 2	1.000	2 X 3	0.841
CAL-2	1.98	1.80	2.88		1 X 3	0.841	2 X 4	<0.001
CAL-3	1.95	1.80	2.82		1 X 4	<0.001	3 X 4	<0.001
CAL-4	1.79	1.68	1.42		NA	NA	NA	NA

As shown in Table 6, in the melatonin group, there was a significant and consistent decrease in the mean, median, and mean rank of GI from 2.44, 2.41, and 3.95, respectively, before the intervention to 0.69, 0.60, and 1.00, respectively, three months after the intervention (p < 0.001). All the differences between the different time periods were significant. The same pattern can be applied for PD (p < 0.001), but the difference between times 1 and 2 was not significant (p = 0.408). The same is true for CAL where there was a significant (p < 0.001) and consistent decrease of the CAL values except for the difference between reading at time 1 and 2 (p = 1.000).

**Table 6:** Comparing the studied variables at different time periods in the melatonin group. * By related samples Friedman’s two-way ANOVA by ranks. ** By Bonferroni post-hoc test. GI: gingival index; PD: probing depth; CAL: clinical attachment loss.

Variables - Time	Mean	Median	Mean rank	P*	Time	P**	Time	P**
GI-1	2.44	2.41	3.95	<0.001	1 X 2	0.010	2 X 3	0.002
GI-2	2.18	2.08	3.05		1 X 3	<0.001	2 X 4	<0.001
GI-3	1.26	1.18	2.00		1 X 4	<0.001	3 X 4	0.004
GI-4	0.69	0.60	1.00		NA	NA	NA	NA
PD-1	3.37	3.50	3.64	<0.001	1 X 2	0.408	2 X 3	<0.001
PD-2	3.29	3.25	3.36		1 X 3	<0.001	2 X 4	<0.001
PD-3	2.41	2.38	2.00		1 X 4	<0.001	3 X 4	0.004
PD-4	1.79	1.88	1.00		NA	NA	NA	NA
CAL-1	2.08	2.25	3.30	<0.001	1 X 2	1.000	2 X 3	0.008
CAL-2	2.08	2.25	3.30		1 X 3	0.008	2 X 4	<0.001
CAL-3	1.91	2.00	2.39		1 X 4	<0.001	3 X 4	<0.001
CAL-4	1.27	1.38	1.00		NA	NA	NA	NA

As shown in Table 7, in the vitamin C and melatonin group, there was a significant and consistent decrease of the mean, median, and mean rank of GI from 2.33, 2.30, and 3.85, respectively, before the intervention, to 0.59, 0.50, and 1.00, respectively, three months after the intervention (p < 0.001). All the differences between the different time periods were significant. There was also a significant and consistent decrease in PD values (p < 0.001) but the difference between time 1 and 2 readings was not significant (p = 0.317). The same pattern is observed regarding CAL, but there was no difference between readings 1 and 2 (p = 1.000).

**Table 7:** Comparing the studied variables at different time periods in the vitamin C and melatonin group. * By related samples Friedman’s two-way ANOVA by ranks. ** By Bonferroni post-hoc test. GI: gingival index; PD: probing depth; CAL: clinical attachment loss.

Variables - Time	Mean	Median	Mean rank	P*	Time	P**	Time	P**
GI-1	2.33	2.30	3.85	<0.001	1 X 2	0.021	2 X 3	0.002
GI-2	2.11	2.20	3.08		1 X 3	<0.001	2 X 4	<0.001
GI-3	1.28	1.08	2.07		1 X 4	<0.001	3 X 4	0.001
GI-4	0.59	0.50	1.00		NA	NA	NA	NA
PD-1	3.06	3.25	3.67	<0.001	1 X 2	0.317	2 X 3	<0.001
PD-2	2.94	3.00	3.33		1 X 3	<0.001	2 X 4	<0.001
PD-3	1.72	1.50	1.97		1 X 4	<0.001	3 X 4	0.005
PD-4	1.10	1.00	1.03		NA	NA	NA	NA
CAL-1	2.66	3.00	3.47	<0.001	1 X 2	1.000	2 X 3	<0.001
CAL-2	2.66	3.00	3.47		1 X 3	<0.001	2 X 4	<0.001
CAL-3	2.23	2.25	2.07		1 X 4	<0.001	3 X 4	0.001
CAL-4	0.79	0.88	1.00		NA	NA	NA	NA

## Discussion

Periodontal disease involves inflammation leading to bone and ligament breakdown by osteoclasts [11]. This study found that intra-pocket melatonin gel with vitamin C solution, as supportive therapy for SRP, significantly improved periodontal treatment outcomes and can be considered an innovative addition to treatment methods for periodontal disease.

Oxidative stress plays a crucial role in periodontal disease and SRP presents a limitation in such cases and has provided logic for using pharmacological agents adjunctively to mechanical therapy and antioxidants [27]. Additionally, many studies showed that supplemental treatment with antioxidants resulted in improved clinical periodontal findings, increased activities of local and systemic antioxidants, and decreased levels of local and systemic reactive oxygen species (ROS) when compared with non-surgical periodontal therapy alone [28]. However, in localized inflammatory conditions, the administration of a high systemic dose is required to reach the area of inflammation, which may leave a negative impact on the general health of the patient. On the other hand, the local introduction of a drug provides the needed amount efficiently [26], with fewer adverse effects, less possibility of developing bacterial resistance, and better compliance [29].

This study was planned to estimate the effect of locally delivered melatonin and vitamin C as adjuncts to non-surgical periodontal therapy on the clinical parameters, among patients with chronic periodontitis. The findings revealed the important role melatonin plays in inhibiting oxidative stress by increasing the level of vitamin C in periodontal tissue through intra-pocket application. This was significantly seen in gradual continuous reduction over time in the degree of GI, PD, and CAL after different time intervals of one week, one month, and three months of the therapy, particularly among the vitamin C and melatonin group if compared with the melatonin group and the placebo group. These findings align with a previous study by Montero et al. [28], who reported that topical application of melatonin has a number of positive effects on periodontal health. Similarly, Al-Abdaly et al. [25] reported the same results in locally delivered vitamin C in the treatment of periodontitis. In contrast, Konecna et al. [30], in their study on patients with periodontitis, asked the patients to rinse the oral cavity before sleep and after brushing for 14 days with a solution containing melatonin without being subjected to NSPT, the results did not show any improvement in periodontitis and did not support the use of melatonin for the treatment of periodontitis.

These results are in accordance with the study of Chitsazi et al. [21] in systemic administration of melatonin and vitamin C with non-surgical treatment of chronic periodontitis. The greatest healing outcomes were observed in the vitamin C and melatonin group. This can be attributed to the synergic effect of melatonin and vitamin C. SRP is the gold standard treatment, and melatonin with vitamin C can be used as an adjunct [21]. In addition, a clear improvement in CAL was recorded after three months of the intervention, which demonstrates the powerful antioxidant action of melatonin. In their study on the systemic administration of melatonin and vitamin C in the treatment of periodontitis, Chitsazi et al. [21] showed the same outcomes.

While this study showed substantial improvements in periodontal indices with adjunctive therapies, the findings contrast with those of Konecna et al., who observed limited effects of melatonin when used without SRP [30]. This discrepancy may be attributed to differences in the mode of delivery (oral rinses vs. localized gels) and the absence of mechanical debridement in their study. These variations underscore the importance of combining mechanical and pharmacological approaches for effective periodontal therapy.

The triple-blind design ensured an unbiased assessment of outcomes, and the localized delivery of melatonin and vitamin C minimized systemic side effects while targeting the affected sites efficiently. The study provides robust evidence supporting the adjunctive use of these agents in improving clinical parameters such as GI, PD, and CAL.

The primary drawback of the study was the recruitment of participants with chronic periodontitis without any systemic diseases. The follow-up challenge was also another limitation of the study. To cover the loss of follow-ups, we increased the sample size. However, strict adherence to inclusion and exclusion criteria minimized confounding factors, enhancing the reliability of the results. Furthermore, a representative sample was guaranteed by the use of basic random sampling, enhancing the validity of the results.

## Conclusions

Based on the results of the current study, treatment with melatonin and vitamin C as an adjunct to SRP may improve periodontal indices (PD, CAL, and GI) compared to SRP alone. Combined use of these two locally delivered drugs showed better improvement in the long term.
